# Sarcomatoid Renal Cell Carcinoma: Biological Features and Therapeutic Implications—A Narrative Review

**DOI:** 10.1007/s11934-026-01325-4

**Published:** 2026-03-07

**Authors:** Francesco Lasorsa, Martina Milella, Monica Rutigliano, Antonio di Bari, Antonio d’Amati, Savio Domenico Pandolfo, Gabriele Bignante, Alessandro Caniglia, Riccardo Autorino, Pasquale Ditonno, Giuseppe Lucarelli

**Affiliations:** 1 Department of Precision and Regenerative Medicine and Ionian Area, Urology and Kidney Transplantation Unit, University of Bari “Aldo Moro”, Bari, Italy; 2 Anatomical Pathology Unit, Fondazione Policlinico Universitario ″A. Gemelli″ IRCCS, Università Cattolica S. Cuore, Rome, 00168 Italy; 3 Department of Medicine and Surgery, University LUM Giuseppe Degennaro, Casamassima, Bari, Italy; 4 Department of Neurosciences, Reproductive Sciences and Odontostomatology, University of Naples Federico II, Naples, Italy; 5 Department of Urology, University of L’Aquila, L’Aquila, Italy; 6 Division of Urology, Department of Oncology, University of Turin, San Luigi Gonzaga Hospital, Orbassano, Turin, Italy; 7 Pathology Unit, IRCCS Istituto Tumori “Giovanni Paolo II”, Bari, 70124 Italy; 8 Department of Urology, Cleveland Clinic, Cleveland, OH USA; 9 Urology Unit, IRCCS Istituto Tumori “Giovanni Paolo II”, Bari, Italy

**Keywords:** Sarcomatoid, Renal cell carcinoma, Metabolism, Pathology, Treatment

## Abstract

**Purpose of the Review:**

This review underscores the importance of recognizing sarcomatoid features in renal cell carcinoma, as this component significantly impacts prognosis and treatment strategies.

**Recent Findings:**

Sarcomatoid renal cell carcinoma (sRCC) is a rare and aggressive form of renal cancer characterized by poor prognosis and distinct biological features. Despite its clinical significance, there remains a knowledge gap regarding the comprehensive understanding of its molecular and therapeutic landscape. Recent studies provided an extensive overview of the clinical, pathological, and molecular characteristics of sRCC, highlighting its unique genomic alterations and immune profile. Notably, sRCC exhibits a highly inflamed tumor microenvironment, which correlates with increased responsiveness to immune checkpoint inhibitors.

**Summary:**

Our findings suggest that integrating immunotherapy into the management of sRCC could enhance patient outcomes, paving the way for future research and clinical applications in this challenging cancer subtype.

## Introduction

Renal cancers are estimated to be diagnosed in 80,980 people and to contribute to 14,510 deaths in the United States in 2025 [1]. Renal cell carcinoma (RCC) is the most common solid lesion of the kidney and accounts for more than 90% of all kidney cancers, with clear cell RCC (ccRCC) representing the predominant histological subtype. The incidence peaks between the ages of 60 and 70 years, and the male-to-female ratio is 1.5:1 [2].

**Fig. 1:**
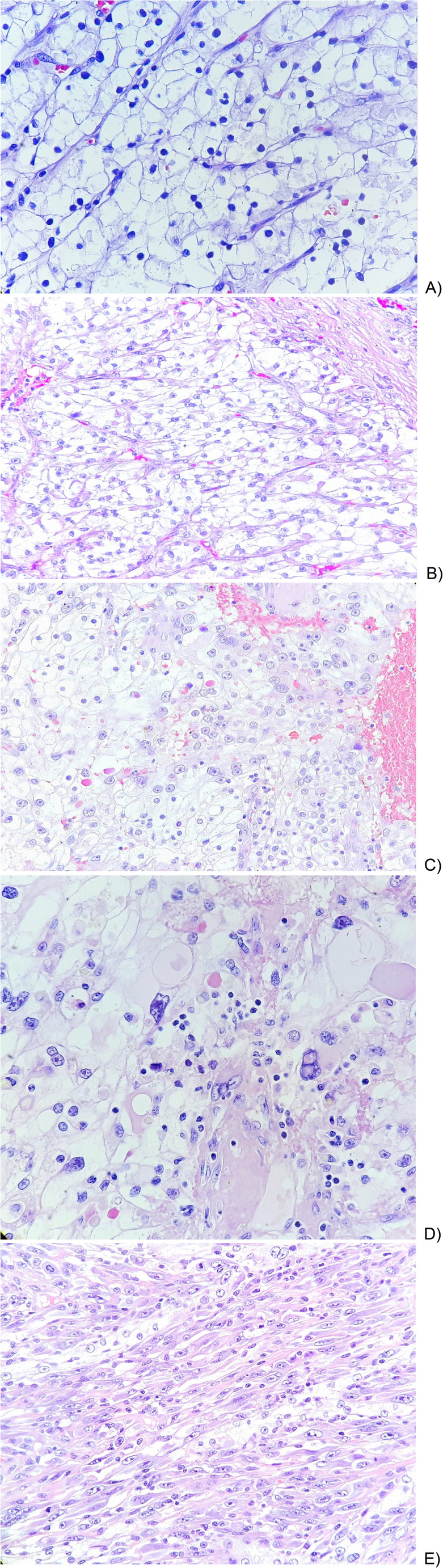
WHO/ ISUP grade classification. (**A**) Grade 1; (**B**) Grade 2; (**C**) Grade 3; (**D**) Grade 4; (**E**) Sarcomatoid RCC

In 2022, the 4-tier World Health Organization (WHO)/ International Society of Urological Pathology (ISUP) updated the Fuhrman grade classification (Fig. 1) [3]. High-throughput analyses revealed distinct, grade-dependent molecular and metabolic alterations in ccRCC, underscoring their potential as targets for future therapeutic strategies [4, 5].

Sarcomatoid feature is no longer regarded as an independent histological subtype of RCC. Instead, sarcomatoid dedifferentiation can emerge within various established RCC histologies, including clear cell, papillary, and chromophobe RCC, collecting duct carcinoma, thyroid-like follicular carcinoma, acquired cystic disease–associated RCC, mucinous and tubular spindle cell carcinoma, ALK-rearranged RCC, succinate dehydrogenase–deficient RCC, and malignant mixed epithelial and stromal tumor [6]. ccRCC and chromophobe RCCs were the most common entities.

RCCs that undergo sarcomatoid dedifferentiation are generally classified as sarcomatoid RCCs (sRCCs). Sarcomatoid features are observed in approximately 4–5% of all RCCs. These tumors are often diagnosed at an advanced or metastatic stage (~ 20–40% of sRCC are localized), and this cohort of patients has a survival rate of less than 1 year [7]. Compared with non-sarcomatoid RCC, sRCC is associated with poorer survival outcomes across all disease stages [8]. A higher percentage of sarcomatoid features worsens the patient prognosis. A recent study demonstrated that the sarcomatoid component emerged as an independent factor for cancer-specific survival (CSS) and recurrence-free survival (RFS) in non-metastatic sarcomatoid ccRCC [3]. Patients with a low sarcomatoid percentage (< 20%) had a markedly longer CSS than those with a high sarcomatoid one (41 vs. 16 months, *p* < 0.0001). The median RFS also differed significantly between the two groups (32 vs. 11 months, *p* < 0.0001) [3].

Metastatic lesions derived from sRCC typically show either 0% or 100% sarcomatoid involvement, which is consistent with metastasis arising from a single tumor cell clone. In primary tumors, a sarcomatoid component greater than 30% significantly increases the probability of sarcomatoid morphology in metastases [9].

The aim of this narrative review is to summarize the current knowledge on the clinical, pathological, and molecular characteristics of sRCC, and to provide an updated overview of contemporary approaches to their management.

## Tumor Biology

The most frequently enriched genetic alterations involve TP53, BAP1, and NF2 in sRCC, similar to other solid cancer [10]. Distinctive genomic features include deep deletions of CDKN2A and CDKN2B, and amplification of EZH2, MYC, and CCNE1. Increased expression of cell cycle and proliferation (CCNB1, CDC45, CDC6, CDCA3, CDCA7, CDCA8, CDK6, and MKI67), immune (HIVEP3, IFI16, IFI35, IL15RA, and LAG3), and metastasis-implicated genes (ACTB, ANLN, ARPC1B, ARPC5, and ARPC5L, CD44), chemokines (CXCL9), and antigen-presenting machinery (TAP1, TAP2, CALR, PSMA5, PSMB10, PSMB4, PSMC2, and PSME2) genes were revealed by genomic analysis [11]. After dedifferentiation, markers of the hypoxia-inducible factor pathway (HIF-1α, CAIX, GLUT1, VEGF) continue to be expressed [12].

Sarcomatoid dedifferentiation relies on a process known as epithelial-to-mesenchymal transition (EMT) [13]. In RCCs, epithelial cells lose their epithelial features and gain mesenchymal traits. Multiple signaling pathways may drive EMT, including TNF, TGFβ, Wnt, MAPK, and PI3K/AKT [14]. This results in the subsequent activation of several transcription factors, such as Snail, Zeb, and Twist, thus leading to the downregulation of epithelial markers (E-cadherin) and upregulation of mesenchymal markers (N-cadherin, vimentin, and S100A4) [15]. In this scenario, epigenetic mechanisms are thought to contribute to EMT [16].

Transcriptomic differences emerge when stratifying sRCC based on the abundance of sarcomatoid features [17]. Sarcomatoid-high tumors (≥10% sarcomatoid features) showed abnormalities in cell-cycle control and proliferation, which may explain their rapid growth (enrichment of the G2M checkpoint, E2F targets, and mitotic spindle sets) [17]. These cells exhibit redox changes, suggesting altered ferroptosis. These features are typical of aggressive, treatment-resistant tumors and align with previous findings of BAP1 loss and iron depletion in sRCC compared to non-sarcomatoid RCC. BAP1 loss promotes ferroptosis escape by increasing antioxidant production, thus preventing iron-dependent oxidative stress–induced regulated cell death [18]. Hypoxia and hypoxia-implicated TNF-α pathway enriched sarcomatoid-low RCCs (< 10% sarcomatoid features) [17, 19].

In addition, transcriptomics and immunohistochemical evaluation confirmed an immune-inflamed phenotype of sRCC because of its increased CD8 + T cell infiltration, CD8+/CD4 + T cell ratio, activated/resting NK cell ratio, M1 macrophages, M1/M2 macrophage ratio, and Th1 score. Taken together, these features may explain the high responsivity of sRCC to immune checkpoint inhibitors (ICIs) [11].

## Metabolic Landscape

Metabolic reprogramming is increasingly being recognized as a critical factor in the pathogenesis and progression of RCC, and recent studies have highlighted the complex interplay between tumor metabolism and its microenvironment [20, 21].

In RCC, this reprogramming is characterized by shifts in nutrient utilization, such as a preference for aerobic glycolysis (the Warburg effect), lipid anabolism, and glutamine metabolism [22, 23]. These metabolic changes not only support tumor growth and proliferation but also contribute to immune evasion and therapeutic resistance [24, 25].

Previous studies, which used a combined proteomic and metabolomic analysis of RCC tissues and primary cell cultures of various grades, revealed that higher-grade tumors showed a more pronounced Warburg effect, a lower reliance on the tricarboxylic acid cycle, and changes in fatty acid and glutamine metabolism. These studies also identified a significant grade-dependent increase in tryptophan catabolism linked to immunosuppression [26–29]. Sarcomatoid RCCs are characterized by differences in their transcriptomic profiles compared with non-sarcomatoid RCCs [30–32]. A progressive decrease in the expression of HIF-1α has been shown in ccRCC progression from low-grade to high-grade tumors with abundant sarcomatoid components. In contrast, Myc expression is higher in sRCC than in low-grade tumors [5]. This differential modulation of the expression of transcription factors translates into a different metabolic profile in relation to the percentage of sarcomatoid components. In fact, tumors with a high abundance of sarcomatoid components exhibited increased accumulation of the glycolytic intermediates, glutamate and glutamine, whereas high-grade tumors with a low percentage of sarcomatoid elements had increased levels of citrate and fatty acids, suggesting that metabolic reprogramming differs between these two subtypes of high-grade RCC [5].

## Pathology

### Definition and Classification Framework (WHO 2022)

Sarcomatoid differentiation is currently regarded as a high-grade dedifferentiation pattern rather than a distinct histologic subtype and may arise in virtually any recognized RCC entity. According to the most recent WHO classification of tumors of the urinary and male genital systems, the diagnosis should specify the underlying epithelial RCC subtype, when identifiable, and the estimated proportion of sarcomatoid components, given its strong prognostic and clinical relevance [33]. Even minimal sarcomatoid changes are sufficient to classify tumors as sarcomatoid RCCs [34]. Conversely, purely sarcomatoid tumors should be referred to as RCC, NOS [35]. 

### Gross Pathology

On gross examination, sRCCs are typically large (median size, 10 cm) and heterogeneous, with a firm, fleshy, grey-to-white cut surface. Extensive tumor necrosis and hemorrhage are common and often reflect rapid growth and high-grade biology. In nephrectomy specimens, the sarcomatoid component frequently corresponds to solid, tan-white areas, which is distinct from the more typical appearance of the epithelial RCC component [36]. 

### Histopathology

Microscopically, sRCC usually show a biphasic architecture composed of an epithelial RCC component and a malignant sarcoma-like component, although the latter may predominate or even appear exclusively in limited samples. The sarcomatoid component most commonly consists of high-grade spindle cells arranged in intersecting fascicles (fibrosarcoma-like pattern); however, pleomorphic undifferentiated sarcoma–like morphology is also frequently encountered. Marked nuclear atypia, brisk mitotic activity, and extensive coagulative necrosis are typical features [6, 37]. Less commonly, heterologous differentiation may be observed, including osteosarcomatous, chondrosarcomatous, rhabdomyosarcomatous, angiosarcomatous, or liposarcomatous-like elements. These findings should be explicitly reported as they further highlight the dedifferentiated nature of the tumor and may complicate differential diagnosis [6]. Sarcomatoid RCC is often associated with adverse pathological features, including renal sinus invasion, renal vein involvement, extrarenal extension, lymphovascular invasion, and lymph node metastasis. Importantly, sarcomatoid changes may be focal, underscoring the need for extensive sampling of morphologically heterogeneous areas in resection specimens to accurately identify the epithelial component and estimate the sarcomatoid proportion [7, 37].

### Immunohistochemistry

Immunohistochemistry plays a crucial role in establishing the renal epithelial lineage and excluding relevant morphological mimics, including sarcomatoid urothelial carcinoma, angiomyolipoma, dedifferentiated liposarcoma, sarcomatoid adrenocortical carcinoma, and primary mesenchymal neoplasms. This approach is particularly critical in cases lacking an identifiable epithelial component and in small biopsy specimens dominated by spindle- or pleomorphic cells [7]. Markers of renal epithelial differentiation, notably PAX8 (and less consistently PAX2), are frequently retained in sarcomatoid areas, although their expression may be focal or weak. Broad-spectrum cytokeratins and EMA may also be focally positive, but their absence does not exclude sarcomatoid RCC, reflecting epithelial–mesenchymal transition and dedifferentiation. Vimentin is commonly expressed but lacks specificity [7, 38]. When a clear cell RCC background is suspected, CAIX and CD10 may be helpful as they are often retained even within the sarcomatoid regions. However, interpretation should always be integrated with morphology and the presence of an epithelial component, when available [7]. A targeted exclusion panel is essential for the differential diagnosis of spindle-cell renal tumors. Melanocytic markers (HMB45 and MelanA) are typically negative in sRCC, helping to exclude PEComa/angiomyolipoma. Desmin and other myogenic markers are generally absent in sarcomatoid areas, aiding the distinction from primary renal smooth muscle neoplasms. In cases involving the renal pelvis or raising concerns for sarcomatoid urothelial carcinoma, urothelial markers such as GATA3 and p63/p40 may be informative in the appropriate anatomic and clinical context [6, 7, 38].

Several studies have demonstrated increased PD-L1 expression and tumor-infiltrating lymphocytes in sRCC compared to non-sarcomatoid RCC, reflecting an immune-inflamed tumor microenvironment [39, 40]. Zhao et al. recently reported that PD-L1 expression is associated with larger tumor size and pathologic T stage in sRCC. The PD-L1 positive subgroup experienced shorter OS, whereas a higher tumor stage was the only independent prognostic factor for unfavorable OS in the PD-1 positive subgroup of sRCC [41]. In the CheckMate 214 trial, sRCC showed higher PD-L1 expression and derived a marked clinical benefit from immune checkpoint inhibition with nivolumab plus ipilimumab [42]. Although not diagnostic, these findings are biologically relevant and support the integration of pathology with therapeutic decision-making.

### Molecular Biology

At the molecular level, sarcomatoid and rhabdoid differentiation in RCC is associated with genomic and transcriptional features of aggressive behavior, rather than with a unique pathognomonic alteration. Recurrent molecular events include alterations affecting cell cycle regulation and chromatin remodeling, such as *CDKN2A/B* loss and *BAP1* inactivation, which are enriched in sarcomatoid and rhabdoid tumors across RCC subtypes. These alterations are thought to contribute to dedifferentiation, genomic instability, and rapid tumor progression [43, 44]. Transcriptomic analyses have shown the activation of mesenchymal and *MYC*-related transcriptional programs, consistent with epithelial–mesenchymal transition and high-grade morphology [11]. In parallel, sRCCs display molecular signatures associated with immune activation, including interferon-γ-related pathways and increased expression of immune checkpoint molecules, correlating with high PD-L1 expression and immune infiltration observed histologically [45]. Together, these molecular features reinforce the concept of sarcomatoid change as a terminal dedifferentiation state, with both aggressive biological behavior and relative immunogenicity.

### Differential Diagnosis

When only the sarcomatoid component is represented, particularly in the biopsy material, the differential diagnosis includes primary renal sarcomas, PEComa/epithelioid angiomyolipoma, and sarcomatoid urothelial carcinoma of the upper urinary tract. Careful integration of morphology, immunohistochemistry, radiologic findings, and tumor epicenter is essential [6]. The differential diagnostic features and immunophenotypic profiles of these entities are summarized in Table 1. 

**Table 1:** IHC markers and main differential diagnoses of sRCC

Entity	Positive IHC markers	Negative IHC markers
Sarcomatoid renal cell carcinoma	PAX8 / PAX2; EMA (often focal); pan-cytokeratin (often focal); CAIX (particularly in ccRCC-derived cases); vimentin	SMA; desmin; HMB45; Melan-A
Leiomyosarcoma	SMA; desmin; h-caldesmon	PAX8; pan-cytokeratin; EMA; CAIX
Angiomyolipoma / PEComa (epithelioid variant)	HMB45; Melan-A; SMA	PAX8; pan-cytokeratin; EMA; CAIX
Mucinous tubular and spindle cell carcinoma (MTSCC)	PAX8; CK7; AMACR	CAIX; melanocytic markers (HMB45, Melan-A)
Sarcomatoid urothelial carcinoma	GATA3; pan-cytokeratin; EMA; p63/p40	PAX8; CAIX

*EMA* epithelial membrane antigen, *CAIX* carbonic anhydrase IX, *SMA* smooth muscle actin, *CK* cytokeratin, *MTSCC* mucinous tubular and spindle cell carcinoma, *AMACR*  alpha-methylacyl-CoA racemase

### Reporting Recommendations

Given its major prognostic and therapeutic implications, sarcomatoid differentiation should be explicitly reported in all RCC specimens. Structured pathology reports should include the estimated percentage of sarcomatoid components, the underlying RCC subtype, the presence of rhabdoid features, and associated adverse parameters such as necrosis, lymphovascular invasion, renal sinus or vein invasion, extrarenal extension, margin status, and pathological stage [46].

## Treatment

### Surgical Management

The role of cytoreductive nephrectomy (CN) in patients with sRCCs remains controversial. sRCCs are usually large primary tumors or bulky diseases at initial presentation. Therefore, removing the large primary mass has been speculated to affect the response to subsequent immunotherapy [47].

Approximately 77–80% of patients have been reported to recur within 5–26 months after radical nephrectomy with curative intent [48].

A retrospective analysis compared partial nephrectomy (PN) to radical nephrectomy (RN) for the treatment of sRCC (T1-T3 N0- N1M0) from the National Cancer Database. Compared to RN, PN was associated with improved overall survival in pT1 (85.5% vs. 72.8%, *p* < 0.001) and pT3 (68.2% vs. 47.2%, *p* < 0.001), but not in pT2 (77.6 vs. 66.7%, *p* = 0.136) [49].

Moreover, a similar median OS was found in a propensity-score matching analysis for cT1N0M0 sRCCs (132 vs. 100 months, *p* = 0.11) [50].

Systemic treatment may be preceded by CN in case of metastatic disease.

The median disease-specific survival was 7 months (IQR 3–17) among patients who underwent cytoreductive nephrectomy for metastatic sRCCs, compared to 4 months (IQR 2–7) in those who did not. As such, CN emerged as an independent factor for improved disease-free survival (HR 0.53, 95% CI 0.43–0.66, *p* < 0.001) [51].

Consistent with these findings, CN significantly improved OS (HR 0.57, 95% CI 0.35–0.93) in patients with metastatic sarcomatoid and/or rhabdoid RCC treated with immune checkpoint inhibitors (ICIs) [52].

A recent multicenter study explored the optimal timing of CN in mRCC, highlighting a clear survival benefit in patients without sarcomatoid features [53]. Improved survival outcomes were observed in patients without sarcomatoid tumors (HR 0.46, 95% CI 0.37–0.57). In patients with sRCC, the median OS was 24 months when CN was performed after systemic therapy and 36 months when CN preceded systemic therapy. However, this difference was not statistically significant (upfront CN: HR 1.10, 95% CI 0.70–1.73). Conversely, upfront CN was associated with higher complete response rates (5.3% vs. 0%, *p* = 0.42) [53]. Therefore, the authors speculate that an upfront CN before systemic treatment might be a favorable treatment sequence for metastatic sRCC.

### Systemic Therapy

Cytotoxic chemotherapy yielded poor survival outcomes in sRCCs, with OS never reaching > 9 months and disease progression occurring within 4 months. Response rates in sRCC remain poor, even when using two otherwise effective standard RCC treatments (gemcitabine+ sunitinib and capecitabine+gemcitabine+bevacizumab) [7].

Blum et al. reported the preliminary results of trials assessing ICIs at the time of their study [7]. Compared with sunitinib, ICIs demonstrated higher objective response rates (ORR) and an approximately 40% reduction in the risk of progression and death in those abstracted results [54–56]. Evidence showing high expression of the PD-1/PD-L1 axis and CTLA-4 on regulatory T cells provides further rationale for these promising ongoing trials [39, 57, 58].

Table 2 summarizes the most updated results of systemic treatment for sRCCs.

**Table 2:** Targeted therapy for treatment of sRCC

	Treatment	*n*	Response rate	Outcomes
Karam et al.(NCT00326898) [59]	SunitinibSorafenib	5258	NR	5-yr DFS: 33.6%5-yr OS: 51.8%5-yr DFS: 36.0%5-yr OS: 55.9%
Rini et al. (IMmotion151) [56]	Atezolizumab + Bevacizumab Sunitinib	68 74 (mRCCs)	CR: 10% CR: 3%	Median PFS: 8.3 months Median OS: 21.7 months Median PFS: 5.3 months Median OS: 15.4 months
Choueiri et al. (JAVELIN Renal 101) [60]	Avelumab + axitinib Sunitinib	47 61	CR: 4.3% CR: 0%	Median PFS: 7.0 months 12-mo OS: 83.0% Median PFS: 4.0 months 12-mo OS: 67.0%
Tannir et al. (CheckMate 214) [54]	Nivolumab + ipilimumab Sunitinib	74 65	CR: 18.9% CR: 3.1%	Median PFS: 26.5 months Median PFS: 5.1 months
Ciccarese et al. (ARON-1 study) [65]	IO + IO IO + TKI	125 (77 mRCCs) 101 (66 mRCCs)	ORR: 48% ORR: 48%	Median OS: 26.4 months Median PFS: 12.3 months Median OS: 34.4 months Median PFS: 12.4 months
Chahoud et al. [68]	Nivolumab Nivolumab + Ipilimumab Pembrolizumab Avelumab + axitinib Nivolumab + bevacizumab Tremelimumab	20 17 3 1 6 1 (mRCCs)	ORR: 35.4% CR: 16.7%	Median PFS: 4.9 months Median OS: 28.4 months
Kucharz et al. [66]	Cabozantinib	16	NR	Median PFS: 8.8 months Median OS: 11.9 months
Santoni et al. [67]	Cabozantinib (II line)	66 (mRCCs)	NR	Median PFS: 7.59 months Median OS: 9.11 months
Hahn et al. [69]	Cabozantinib Other VEGFR TT Lenvatinib + everolimus ICT + TT	16 7 2 4 (mRCCs after ICI)	NR	Median OS: 23.8 months
Urman et al. [71]	TKI IO IO + IO IO + TKI	19 1 3 2	NR	Median OS: 43.4 months
Sawaya et al. [70]	TT IO	152 91	CR: 1.3% ORR: 13.8% CR: 12.1% ORR: 49.5%	Median OS: 18 months Median OS: 48 months
Grünwald et al. (CLEAR) [64]	Lenvatinib + Pembrolizumab Sunitinib	17 5	ORR: 60.7% CR: 10.7% ORR: 23.8% CR: 0	Median PFS: 11.1 months Median PFS: 5.5 months
Motzer et al. (CheckMate 9ER) [63]	Nivolumab + Cabozantinib Sunitinib	34 41	ORR: 55.9% ORR: 22.0%	Median PFS: 10.9 months Median PFS: 4.2 months
Choueiri et al. (KEYNOTE-564) [61]	Pembrolizumab Placebo	52 59	NR	OS HR: 0.69 (0.28–1.70)

*NR* not reported, *CR* complete response, *ORR* objective response rate, *DFS* disease-free survival, *OS* overall survival, *PFS*  progression-free survival

A post hoc analysis from Eastern Cooperative Oncology Group-American College of Radiology Imaging Network (ECOG-ACRIN) E2805 included 171 patients with sarcomatoid features receiving adjuvant sunitinib, sorafenib or placebo. The 5-year DFS rate was 33.6%, 36.0%, and 27.8%, respectively. Therefore, adjuvant sunitinib or sorafenib did not improve neither DFS (HR 0.74, 95% CI 0.45–1.20 for sunitinib vs. placebo; HR 0.82, 95% CI 0.53–1.28 for sorafenib vs. placebo) nor OS [59].

A subgroup analysis from the IMmotion151 Clinical Trial assessed atezolizumab plus bevacizumab versus sunitinib for untreated metastatic RCCs and sarcomatoid features (68 vs. 74 patients). The rates of objective (49% vs. 14%) and complete response (10% vs. 3%) were larger in the atezolizumab + bevacizumab group, so as the median OS (21.7 vs. 15.4 months) [56].

Avelumab plus axitinib versus sunitinib for sRCCs was evaluated in a post hoc analysis from the randomized JAVELIN Renal 101 trial [60]. Sarcomatoid histology was found in 108 patients: 47 in the avelumab + axitinib group and 61 in the sunitinib group. Avelumab + axitinib was associated with a higher objective response (46.8% vs. 21.3%), complete response (4.3% vs. 0%), and better progression-free survival (HR 0.57, 95% 0.325–1.003). Next, gene signatures were used with sRCC with elevated gene expression of CD274 (PD-L1 gene), CD8A, IFNG, and FOXP3, and reduced VEGF signaling pathway molecules (FLT1 and KDR). In contrast to non-sarcomatoid samples, sRCCs were found to be enriched in a variety of immune cell populations, including activated and resting CD4 memory cells, follicular helper T cells, CD8 T cells, activated natural killer cells, M1 and M2 macrophages, gamma-delta T cells, Treg cells, and activated dendritic cells [60].

The efficacy of nivolumab plus ipilimumab versus sunitinib was investigated in a post hoc analysis of the CheckMate 214 trial in intermediate-poor sRCCs. Overall, 74 and 65 patients were treated with nivolumab + ipilimumab and sunitinib, respectively. Nivolumab + ipilimumab offered higher rates of objective response (60.8% vs. 23.1%) and complete response (18.9% vs. 3.1%). The OS HR was 0.45 (95% CI, 0.3–0.7; *P* = 0.0004) for the combination, as well as the PFS HR 0.54 (95% CI, 0.33–0.86; *P* = 0.0093) [54].

KEYNOTE-564 currently stands alone in providing evidence of both DFS (HR 0.72; 95% CI 0.59–0.87) and OS (HR 0.62; 95% CI 0.44–0.87) benefit with adjuvant pembrolizumab in patients with intermediate- to high-risk RCC [61]. Indeed, adjuvant pembrolizumab is recommended for patients within 12–16 weeks from nephrectomy [62]. In the subgroup analysis, sarcomatoid features were present in 8 and 12 patients receiving pembrolizumab and placebo, respectively (OS HR 0.69, 95% CI 0.28–1.70) [61].

The results of the CheckMate 9ER trial for 2021 were extracted. A total of 34 patients with sRCC were assigned to nivolumab plus cabozantinib and 41 to sunitinib [63]. With a median follow-up of 18.1 months, improved PFS [10.9 vs. 4.2 monhts; HR 0.39 (95% CI 0.22–0.70)], OS [NR vs. 19.7 months; HR 0.36 (95% CI 0.16–0.82)], and ORR (55.9% vs. 22.0%) were observed with nivolumab plus cabozantinib sRCC patients.

In the phase III CLEAR trial, 19 and 16 patients with sarcomatoid features were randomized to receive pembrolizumab plus lenvatinib and sunitinib, respectively (with an additional lenvatinib/everolimus arm). In the sRCC subgroup, the combination achieved higher ORR (60.7% vs. 23.8%), a greater complete response rate (10.7% vs. 0%), and longer PFS (11.1 vs. 5.5 months) than sunitinib [64].

A sub-analysis from the ARON-1 study compared first-line ICI + TKI versus ICI + ICI in metastatic sRCCs. No differences were found in terms of OS (34.4 vs. 26.4 months, *p* = 0.729), and PFS (12.4 vs. 12.3 months, *p* = 0.606). Regardless of the combination strategy, this study outlined the survival benefits of ICI-based combinations in sRCC [65].

Cabozantinib as first-line monotherapy was evaluated in 16 patients with metastatic RCC exhibiting sarcomatoid dedifferentiation, achieving a median PFS of 8.8 months and median OS of 11.9 months [66]. Santoni et al. reported outcomes of cabozantinib administered as second- or third-line therapy in metastatic sRCC, with median PFS and OS of 7.59 and 9.11 months, respectively [67].

ICIs were used to treat metastatic RCC with sarcomatoid dedifferentiation in a single-center study. The overall PFS and OS were 4.9 and 28.4 months. Remarkably, sarcomatoid ccRCC demonstrated a longer PFS (HR 0.25 [95% CI: 0.08, 0.78], *p* = 0.0145) and longer OS (HR 0.13 [95% CI: 0.04, 0.44], *p* = 0.0009) than non-ccRCC [68].

The median OS ranged from 18 to 48 months in cohorts of sRCC patients receiving ICI or TKI monotherapy or their combination [69–71].

## Conclusion

Sarcomatoid dedifferentiation represents an aggressive entity of renal cancer with distinct genetic, epigenetic, and metabolomic profiles.

The therapeutic role of surgery in sRCC remains controversial, largely because of the advanced tumor burden commonly observed at diagnosis. However, the pronounced immunogenic profile of sRCC provides a strong rationale for the use of adjuvant pembrolizumab following curative-intent nephrectomy with the aim of reducing metastatic risk. In metastatic settings, the management of sRCC has evolved substantially in recent years. 

Emerging biological and clinical evidence highlights a highly inflamed tumor microenvironment, which may underlie the distinctive responsiveness to ICI and help explain the survival benefits that were previously difficult to achieve in this aggressive disease. 

## Key References

- Lucarelli G, Lasorsa F, Rutigliano M, Milella M, Spilotros M, d’Amati A, et al. The percentage abundance of sarcomatoid component has a prognostic role in grade 4 non-metastatic clear cell-renal carcinoma. World J Urol. 2025 Apr 23;43(1):243.
  - ○ The abundance of sarcomatoid features has prognostic implications in non-metastatic ccRCC.
- Adeniran AJ, Shuch B, Humphrey PA. Sarcomatoid and Rhabdoid Renal Cell Carcinoma: Clinical, Pathologic, and Molecular Genetic Features. American Journal of Surgical Pathology. 2024 Jul;48(7):e65–88.
  - ○ Continuous research into the sarcomatoid RCC biology is warranted since patients’ prognosis remains poor.
- Choueiri TK, Tomczak P, Park SH, Venugopal B, Ferguson T, Symeonides SN, et al. Overall Survival with Adjuvant Pembrolizumab in Renal-Cell Carcinoma. N Engl J Med. 2024 Apr 18;390(15):1359.
  - ○ Adjuvant pembrolizumab is currently the only treatment shown to provide a survival benefit in patients with intermediate- to high-risk RCC.

## Data Availability

Raw data generated and/or analyzed during the current study are available from the corresponding authors on reasonable request. Authors confirm that figures and tables are original.
